# Emerging Role of Edible Exosomes-Like Nanoparticles (ELNs) as Hepatoprotective Agents

**DOI:** 10.7150/ntno.70999

**Published:** 2022-06-21

**Authors:** P. Debishree Subudhi, Chhagan Bihari, Shiv Kumar Sarin, Sukriti Baweja

**Affiliations:** 1 Department of Molecular and Cellular Medicine, Institute of Liver and Biliary Sciences, Delhi, India.; 2 Department of Pathology, Institute of Liver and Biliary Sciences, Delhi, India.; 3 Department of Hepatology, Institute of Liver and Biliary Sciences, Delhi, India.

**Keywords:** Edible exosome-like nanoparticles (ELNs), Gut microbiome, Liver, Cellular communication and Therapeutics

## Abstract

Liver diseases are responsible for over 2 million deaths each year and the number is rapidly increasing. There is a strong link between edibles, gut microbiota, liver fat and the liver damage. There are very limited therapeutic options for treatment specifically for Alcoholic liver disease (ALD) and Non-Alcoholic liver disease (NAFLD). Recently, identified Edible Exosomes-like nanoparticles (ELNs) are plant derived membrane bound particles, released by microvesicular bodies for cellular communication and regulate immune responses against many pathogens. Many studies have identified their role as hepatoprotective agent as they carry bioactive material as cargoes which are transferred to recipient cells and affect various biological functions in liver. They are also known to carry specific miRNA, which increases the copy number of beneficial bacteria and the production of lactic acid metabolites in gut and hence restrains from liver injury through portal vein. Few in-vitro studies also have been reported about the anti-inflammatory, anti-oxidant and detoxification properties of ELNs which again protects the liver. The properties such as small size, biocompatibility, stability, low toxicity and non-immunogenicity make ELNs as a better therapeutic option. But, till now, studies on the effect of ELNs as therapeutics are still at its infancy yet promising. Here we discuss about the isolation, characterization, their role in maintaining the gut microbiome and liver homeostasis. Also, we give an outline about the latest advances in ELNs modifications, its biological effects, limitations and we propose the future prospective of ELNs as therapeutics.

## Introduction

Liver diseases have pathological spectra ranging from simple steatosis to hepatitis to cirrhosis and hepatocellular carcinoma. Non-alcoholic fatty liver disease is one the most common diet and fat associated non-communicable disease. Presently, 25%-35% and 5%-15% of the general population of Western and Asian countries, respectively, are affected by this disease. This proportion is even higher in people with type 2 diabetes (60%-70%), and in those who are obese or morbidly obese (75%-92%) compared to the general population. Like alcoholic liver disease there is no effective treatment to date for NAFLD. In the absence of a proven effective therapy, we must follow a multi-disciplinary approach in NAFLD treatment. Treatment is mainly directed towards weight loss and risk factor reduction, as most patients are obese or have metabolic syndrome. Dietary modification plays a key role since a carbohydrate-rich diet, especially with high fructose, is the major cause of obesity, insulin resistance and NAFLD development.

Liver disease patients also exhibit gut bacterial overgrowth, enhanced gut permeability and increased paracellular leakage of gut luminal antigens, factors that promote liver damage. A dietary intervention in obese or overweight subjects, consisting of administering an energy-restricted high protein diet during six weeks, increased the diversity of species in the gut, along with decreased adiposity, which reverted to basal levels after the diet was stopped. Dietary components are not only consumed by the host (humans) but also by the gastro-enteric microbiota. The kind of diet we consume effects the microbiota composition as well as the nutritional, toxicological, and biochemical components availability after processing of food in the gut. Though hepatoprotective dietary products have been identified, there is still a need for further investigation to completely understand the mechanism of action of various components in the diet. The major problem is that to attain a certain amount of nutrient, gut microbiota modulation, a large quantity has to be consumed. Hence, a prospective solution to it can be Edible exosomes-Like Nanoparticles (ELNs). ELNs are small membrane vesicles which carry various biomolecules as cargoes 1-3. Hence, they recognized as potential vehicle for intercellular communications in prokaryotes and eukaryotes as well. Natural occurrence of this nano-delivery system in plants are released, mainly to communicate between the cells and regulate the immunity against any pathogenic attack. Plant derived EVs especially from edible portion allows affordable and significant isolation of EVs 4-6. ELNs possesses natural bioactive compounds and non-coding RNAs hence are designed to maintain more stability in facilitating cell to cell communication 7, 8. They have shown efficient and safe delivery of naturally occurring cargoes and artificially loaded cargoes miRNAs 9, and small synthetic drugs 10 proved as promising therapeutic candidates. The properties such as small size, biocompatibility, stability, low toxicity and non-immunogenicity make ELNs as a better therapeutic possibility over other mammalian EVs. ELNs produced by endocytic pathways have many naturally occurring bioactive compounds and non-coding nucleic acids have gained special attention in therapeutics. As isolation of ELNs are preferred from edible food products i.e. fruits and vegetables, many studies *in vitro* and *in vivo* are related to gastrointestinal cell lines or diseases such as inflammatory bowel diseases, gastric cancer, colitis, etc. so far reported.

The liver is one of the vital organs which readily acts as scavenger, contributes in immune surveillance, and is the only organ to have regenerative property. Gut derived products transported into liver by portal vein to liver and in feedback gut receive bile hence forming the interface of gut-liver axis. This interface maintains the gut homeostasis by maintaining the gut microbiota and host immune cells. Mostly in liver diseases this barrier has found disrupted leads to invasion of microbes and microbial particles inside host tissue. There are many therapeutic options which are available for various liver diseases are based on synthetic drugs, prebiotics, probiotics and synbiotics. In this review, we have summarised about ELNs in accordance to their structure, biochemical composition and bio-availabity to target cells, and their promising future therapeutic applications in liver diseases as this could be one of the potent prebiotics which will be safe, efficient and affordable for the treatments.

## Standardised Protocol for Isolation of Edible Exosome-Like Nanoparticles

**Figure 1:**
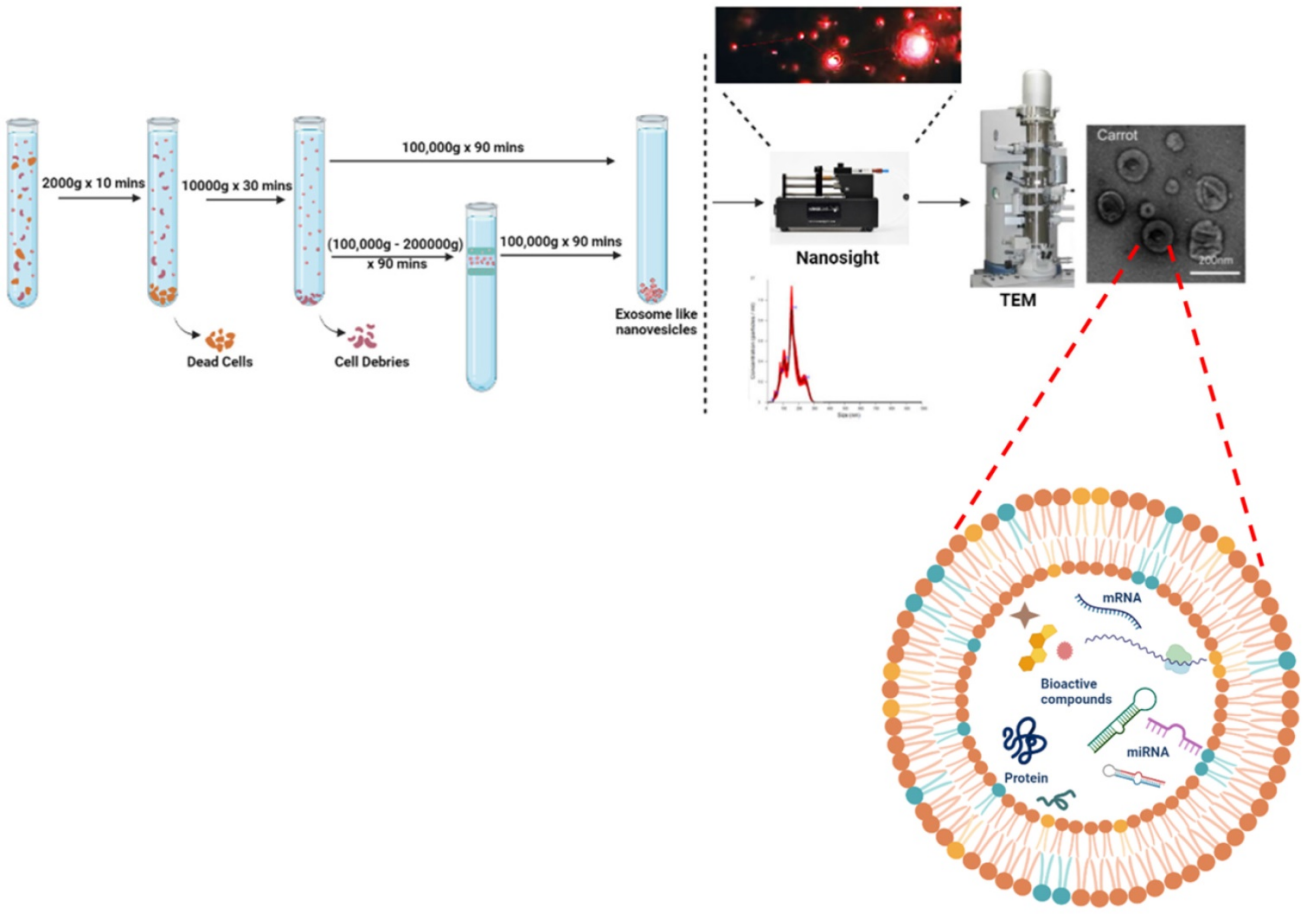
The standardised protocol of ELNs using differential ultracentrifugation and enumeration using nano-sight and electron microscopy. The ELNs charge potential is analysed using zeta potential of membranes.

For isolation of ELNs, there exist various methods, among which differential ultracentrifugation is considered the gold standard. Firstly, edible part of plants are grounded in to juice using mixture then further procedure followed to remove large plant debris or fibres and other unwanted aggregates. Differential ultracentrifugation involves a multistep process that includes low speed centrifugation i.e. 1000 × g to get rid of larger particles or fibres, followed by medium- speed centrifugation of 10,000 × g for removal of larger debris and intact organelles. At last, high-speed ultracentrifugation of 100,000- 150,000 × g was used for pelleting down exosomes or exosome-like particles 11-13. Although the stated methodology gives a high yield production of ELNs but the sometimes there also exists sediments of other vesicles or RNA aggregates as its pelleting efficiency and sensitivity depends on type of rotor (fixed angle or swinging bucket), radius of centrifugal force, angle of rotor sedimentation. Thus another step of gradient ultracentrifugation with extended time of 1- 5 hrs has also been preferred sometimes in order to isolate purified ELNs but with a comprisable yield. Still these methodologies lack the efficient isolation of uniformly sized ELNs. Whereas, there are many approaches available for isolation of mammalian EVs based on ultracentrifugation, precipitation, immuno-affinity, microfluidics and size 14 but for isolation of ELNs ultracentrifugation-based methodology is widely used. However the raised limitation in application of ultracentrifugation mainly due to the need of this specialize equipment and much time as well. Interestingly, another method reported by Yang et al., in which they used a rapid and easy approach, they isolated lemon derived exosomes-like nanoparticles. They combined electrophoresis with that of 300 kDa dialysis bag (approximately of 30 nm pore size) and isolated the ELNs 15. Compared to 4 hours of ultracentrifugation and gradient ultracentrifugation of 5 hours, it was time- saving as it required only 2.5 hours. Although this approach does not involve any special equipment and within short period of time, the biologically active and intact ELNs are isolated but still this technique needs improvement in terms of yield of ELNs. As explained in detail in Fig. 1. To reduce the excessive cost and time in isolation of ELNs, a polymeric precipitation based technique was modified by Kirbas et al. 2019 16 with extra washing steps to reduce phytochemicals present in plant lysates and other contaminants too. They followed the well-established protocol by Shin et al. 17 and Kim et al. 18, they used polyethanol glycol/dextran (PEG/DEX) polymer based Aqueous two-phase system (ATPS), to isolate the ELNs at normal centrifugation speed of 1000g for 10 minutes. In this same line, Kalarikkal et al. used PEG6000 in which PEG form a mesh like net to trap the nanoparticles 19.

## Characterization and Enumeration of Edible Exosome-Like Nanoparticles

Like other mammalian EVs, ELNs can be characterized by various ultrasensitive microscopic methods that are Transmission Electron Microscopy (TEM), Scanning Electron Microscopy (SEM) or Atomic force Microscopy (AFM) and for structural analysis at subcellular level ELNs ultrastructural analysis has been reported by cryo-Electron Microscopy. Where the cup shaped spherical subcellular structures can be seen with their double membranous lipid bilayer and hence identified as ELNs. Characterization of mammalian EVs is also possible by Flow-cytometry as they have specific markers according to their cell of origin. But for ELNs till now there are no specific markers reported, hence this restricts us from using various methods for their characterization. For determination of size and charge of these particles, Dynamic Light Scattering (DLS) has been extensively used. Now days to analyse their size along with concentration Nanoparticle Tracking Assay (NTA) has been followed as it can analyse the Brownian motion of these particles with respective size and concentration. To determine the dispersity or their repulsive nature to avoid aggregation along with stability Zeta analyser can be used to calculate their membrane potential (Fig. 1).

## Uptake, Biodistribution and Bioavailability

The variable surface glycoprotein and lipid compositions among all EVs acts as ligands which are responsible for bio-distribution and specific binding to target cells. Mostly, ELNs uptake had been identified by various ways such as membrane fusion, phagocytosis, macro-pinocytosis and receptor mediated endocytosis. Membrane fusion generally depends on same membrane fluidity of both ELNs and membrane of target cell which requires a low pH 5 20, 21, whereas a neutral pH makes the membrane rigid 22, 23. This suggests that the electrostatic charges between target cell membrane and ELNs influenced by pH could be considered in relation to physiological role of ELNs 24. Whereas, macro-pinocytosis represents a way through which ELNs may deliver their contents to target cells 23, 25, 26. Mostly, uptake of ELNs has been found to occur via phagocytosis 27, 28. Although uptake of ELNs by target cells has not extensively studied but interestingly, it was reported that there is a significant impairment in uptake of ELNs after blocking CD98 receptors in HepG2 cells (hepatoma cell line) suggested a receptor mediated uptake of ELNs 55.

Bio-distribution mainly depends on several factors like parent cell source, uptake mechanism of ELNs by target cells and their retention in circulation along with the membrane composition. Studies of mammalian EVs, RBC-derived EVs showed a gradual uptake by liver (44.9%), bone (22.5%), skin (9.7%), muscle (5.8%), spleen (3.4%), kidney (2.7%) and lung (1.8%). EVs of mammalian cells have specific markers or receptors according to their parent cells which helps them in easy availability to their target cells with the respective ligands for interaction 29, 36, 37, 38. But in case of ELNs the possible parameters reported till date includes the membrane composition and few membrane proteins like lectin II that acts as ligand against CD 98 receptors. The lipid profile of ELNs reveals the presence of galactolipids such as monogalactosyldiacylglycerol (MGDG), digalactosyldiacylglycerol (DGDG), phospholipids that are phosphatidic acid (PA), phosphatidylcholine (PC), phosphatidylethanolamine (PE), other phosphates like 1,2-dilinoleoyl-sn-glycero-3-phosphate (DGP), 1-palmitoyl-2-linoleoyl-sn-glycero-3-phosphate (PLGP), and other lipids in smaller amounts as compared to mammalian exosomes. Different ELNs have their own variation in membrane lipid composition. Turmeric ELNs are rich in PA (34.4%) and DGP whereas Ginger ELNs also possesses the same lipids PA (35.5%) and DGP (36%) or PLGP (34%) in different concentrations. Whereas grapefruit ELNs and garlic ELNs contains PA of 3.5% and 5.5%, respectively of the total lipid content and majorly contains PC, 36.2% and 52.6%, respectively^30^. Among which PA has shown interaction with mammalian target of rapamycin (mTOR) which results in cell growth and proliferation 31 and since it is mitosis phospholipid, and highly fusogenic with calcium presence which induces inter-vesicular fusion 32. Contains high PA composition, garlic ELNs also mostly taken by microglial cells by forming garlic ELN/BASP1 complex, helps in mitigating brain inflammation 33. Based on these studies further question has been raised whether these phospholipids along with their presence and composition are having any role in getting into their target cells. With this the experimental outcomes now confirmed the roles of these phospholipids in uptake of ELNs which are highly rich in PA and were helpful in uptake by *Lactobacilus*, PC- enriched grapefruit derived ELNs and these were preferentially taken up by *Ruminococcaceae* whereas the PA and PC depleted vesicles are not taken up by these bacteria. Considering the *in vivo* bioavailability of this ELNs PA enriched vesicles generally accumulate in gut whereas PC enriched vesicles reach out to liver when given orally. Still the need of understanding the effect of membrane lipid composition in uptake of ELNs by their target cells is open for research.

*In vivo* oral administration of ELNs are well explained by pharmacodynamic study of Acerola ELNs (AELNs) using PKH26 fluorescent dye. They mainly reach out to mice intestine, liver, bladder and ovary after 1hr of consumption 34. A weak fluorescent signal was also found at brain too, which eventually diminished with time. At 6 hrs after administration of Acerola ELNs no signal was detected. With unknown mechanisms or facts intranasal biodistribution of grapefruit derived ELNs has been found in lungs and brain 35, intraperitoneal administration of these ELNs found to be accumulated in liver, spleen, kidney and lung tissues after 72 hrs, whereas intramuscular administration limits their presence in muscles.

## Cargoes of Edible Exosome-Like Nanoparticles

All plant derived EVs have been reported to carry different types of proteins, and nucleic acids as cargos which play a substantial role in intercellular communication. Nucleic acids i.e. miRNAs have been detected. EVs also carries DNA, that can be used for identification of translational biomarkers or mutational modifications in parent cells but their physiological significance is currently unknown.

In 2006, for the first time presence of functional extracellular RNA was reported in murine stem-cell derived EVs 39. These RNAs present inside EVs were predominantly of <700 nts 40, 41. All kind of EVs contain intact mRNAs 42, non-coding RNAs 43, 44, piwi interacting RNA, ribosomal RNA 43, and tRNA fragments 45 in low quantities. However, they have been found enriched with miRNAs (~21 nts) with or without Ago2 protein which believed to increase resistance against RNase 46 and 3'UTR regions having multiple sites for binding of regulatory miRNA so as to play a role in modulating their stability as well as translation 41. Various databases like miRandola, EVpedia or Vesiclepedia have been developed which contains the comprehensive list of miRNAs as cargos with their respective EVs. Extensive studies of miRNAs as cargos have shown various aspects. For example, the action of T-cell derived miR-735 on APCs has shown immune synapse formation 47-49. However, the information on ELNs is still limited and but their known cargoes are tabulated in Table 1. However, the biological relevance of the cargoes in plant derived ELNs are detailed below.

### Effect of Edible Exosome-Like Nanoparticles on Gut

**Figure 2:**
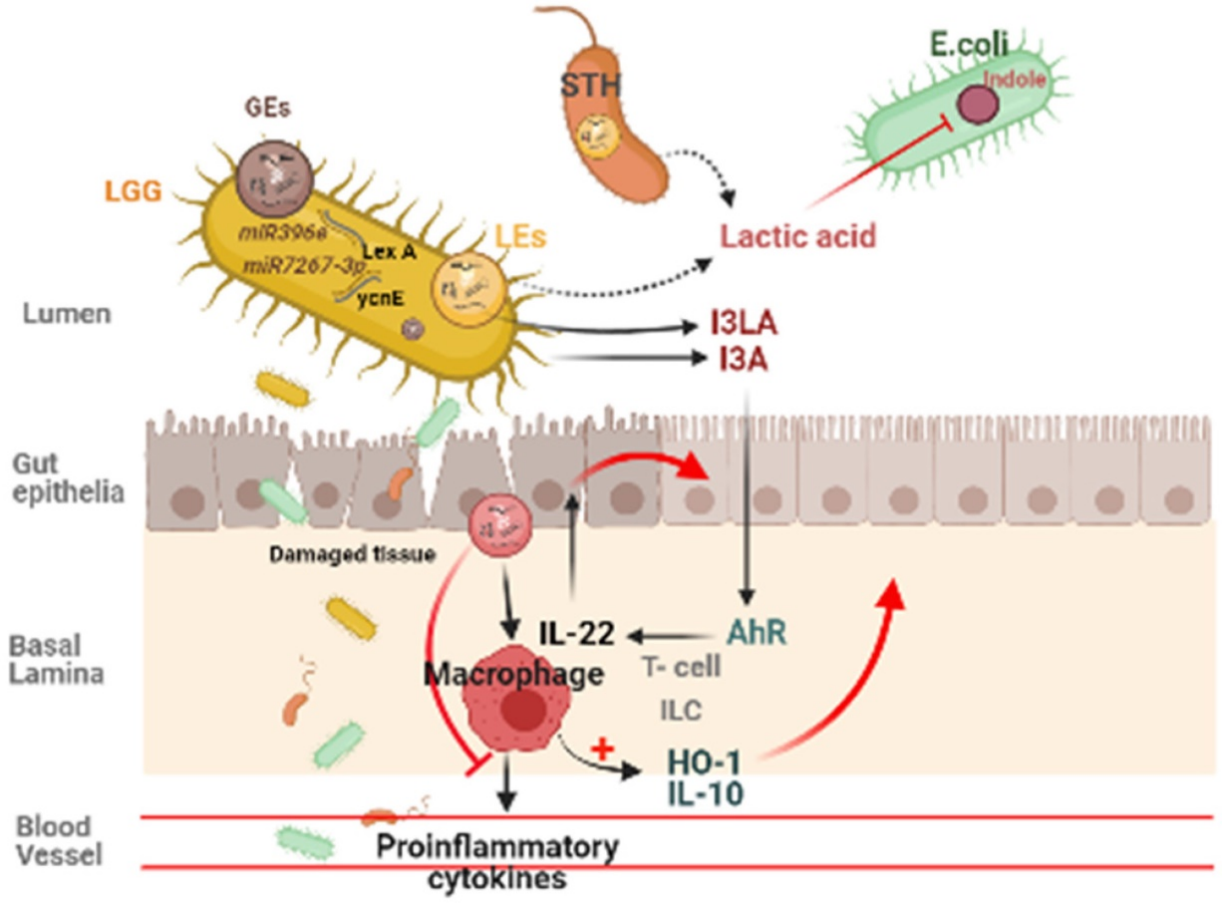
Plant derived ELNs miRNA shapes the gut microbiota homeostasis. ELNs are taken up by gut bacteria and depending on the lipid composition of ELNs, it determines the uptake by specific bacteria, which makes it exclusive growth of lactobacillus production, which effects the production of I3A, which in turn activates the AhR signaling and via IL-22 it inhibits the inflammatory activity in the gut.

Plant derived ELNs are present in our diet and reach the gut with daily intakes. They can also able to withstand the digestive juices and the harsh condition during digestion of food, hence maintains their integrity at gut. Many studies reveals the therapeutic implications of various plant- derived ELNs in mitigating diseases concerned to intestinal barrier and gut microbiota homeostasis such as colitis, inflammatory bowel diseases, colorectal cancer. Ginger derived ELNs carrying miR396e were successfully taken up by *Lactobacillus rhamnassus* GG (LGG) that results in production of lactic acid and its derivatives which acts as ligands to activate anti- inflammatory pathways in host (Fig. 2) 30. At the same time they also possess miR7267-3p which helps in proliferation of LGG in gut. Whereas Lemon derived ELNs enhances bile resistance in LGG by limiting the expression of Msp1 or Msp2 genes and also manipulates *Streptococcus thermophilus* ST-21 (STH) mitigates *Clostridioides difficile* infections (CDI) which is a leading cause of antibiotic resistant colitis 50, 51. A pre- clinical study in diet- induced mice models has shown therapeutic aspects of Orange derived ELNs (OELNs) that mainly targets microsomal triglyceride transfer protein (MTP) and angiopoietin-like protein-4 (ANGPTL4) and reduces the lipid absorption as well as inflammation in gastrointestinal lining. It also did not aggravate steatosis in liver as expression of MTP is high at mRNA level. In addition to this in jejunum the villi size has also been found to be increased after OELNs administration 52. Bovine milk derived extracellular vesicles (BMEVs) could alter the gut microbiota especially the *Clostridiaceae*, *Ruminococcaceae* and *Lachnospiraceae* hence regulate the production of short chain fatty acids (SCFAs) that helps in enhancing intestinal immunity in mice 53. Recently in a study it has been demonstrated that exosomes derived from high fat diet (H-exo) mice, rich in PC can interact with Ahr signalling pathway results into downregulates the expression of hepatic genes i.e. IRS-2. Hence this prevents the activation of PI3K and AKT pathways which results into mitigating insulin resistance 54. From which it can be concluded that not only ELNs with specific lipid composition but also mammalian exosomes with same lipid composition are able to act as the ligand for the same pathway, hence can be transferred from lumen to GI tract and have equivalent therapeutic values. Although ELNs can not only prevent chronic inflammations at gut epithelia but also microglial inflammation. Having the advantage of their small size they also are able to cross the blood brain barrier. Garlic ELNs taken up the microglial cells and inhibits brain inflammation. They help in the downregulation of cMyc gene mediated expression of STING activity which leads to reduce the activities of pro-inflammatory cytokines including IFN-γ and TNF-α 33. Whereas IFN-γ- IDO1 interaction that usually activates the AHR pathway contributes to the attenuation of development of obesity. Hence garlic ELNs metabolites inhibits mitochondrial mediated neuronal cell death and promotes neuronal differentiation contributes to gut-brain axis too.

## Therapeutic Implications of Edible Exosome-Like Nanoparticles on Liver

Many therapeutic studies using ELNs have been reported till date. Most studies explaining the biological significance of ELNs reported against dextran sulphate sodium (DSS) induced colitis, inflammatory bowled diseases and on gut- microbiota. ELNs had shown their potentials like anti-inflammatory, anti- cancerous and anti-oxidant effects by changing the fate of recipient cells. There are few clinical trials which are already registered for liver diseases (Table 2).

**Figure 3:**
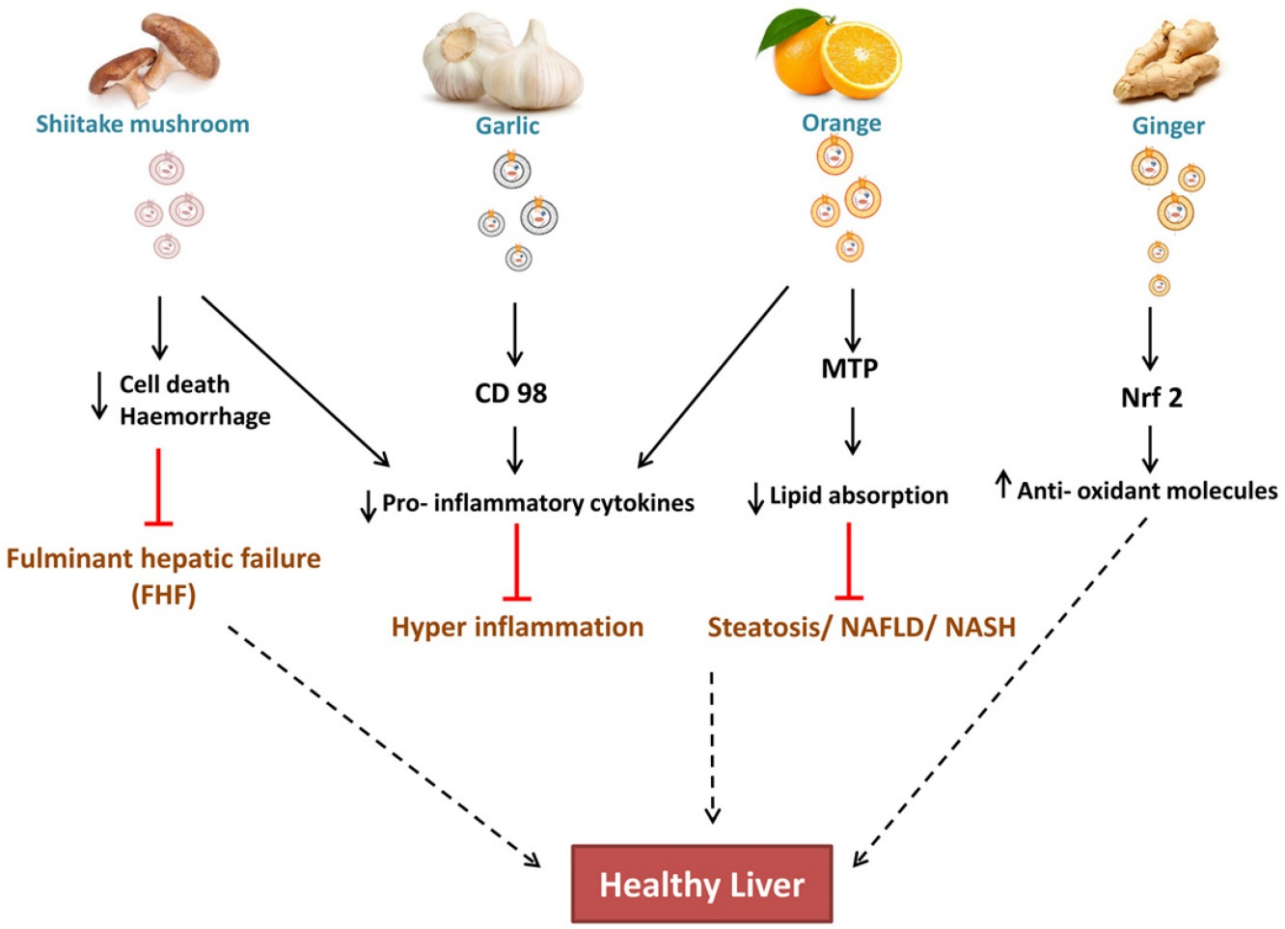
Effect of ELNs like nanoparticles on liver is known to be associated with reducing the hepatocyte damage. As shiitake mushroom ELNs reduces the cell death/haemorrhage and prevents hepatic failure. Garlic ELNs carries CD98 which is anti-inflammatory, orange ELNs prevents lipid absorption and ginger ELNs carries Nrf2 which is an anti-oxidant molecule and contributes in maintaining healthy liver.

Zhuang et al. 2015 reported the hepatoprotective effect of ginger-derived ELNs (GELNs) against alcohol-induced liver damage in mice. They identified Shogaol, the dehydrated analogue of gingerol activates the nuclear factor erythroid 2-related factor 2 (Nrf2) in a TLR4/ TRIF-dependent manner lead to the expression of liver detoxifying/ antioxidant genes like HO-1, NQO1, GCLM, and GCLC. Apart from antioxidant defences, Nrf2 also have critical roles in modulating various cellular processes like hepatocyte proliferation during liver regeneration, inflammation [Nrf2 is involved in maintaining hepatocyte identity during liver regeneration] (Fig. 3) and drug metabolism, hence this finding is also opens up the new avenues for investigating role of ginger ELNs in these respective cellular and molecular mechanisms.

CD98 have a potential role in Non- alcoholic fatty liver diseases (NAFLD), hence co-localization of garlic ELNs indicates to be a promising target for both therapeutic and drug therapy 55. While study of cellular internalization of garlic ELNs interestingly they also observed downregulation of pro-inflammatory factors in HepG2 cells* in vitro*.

In context to metabolic syndrome diseases like hyperlipidaemia, obese- associated intestinal complications, OELNs increases the villi size in jejunum by lowering the fat absorption and chylomicrons production by targeting microsomal triglyceride transfer protein (MTP) hence reduced the plasma lipid concentration. It also acts upon angiopoietin-like protein-4 (ANGPTL4) 27 led to reduced concentration of TNF- α, IL- 1β. Hence OELNs are reported as potential candidates against accumulated plasma lipids or its intake at the level of intestinal barrier, inflammatory intestinal diseases and damaged gut epithelia. Although it is not showing effective reduction of fat in liver steatosis of high- fat, high- sucrose diet mouse models *in vivo*, but due to the reduced absorption of triglycerides at jejunum, helps in systemic reduction of lipid which might lead to amelioration of steatosis. With increase in gene expression of junctional proteins such as CLD1, OCLN, ZO1 it also enhance the recovery of barrier permeability in colitis. Its contribution in concentration of amino acids and bioactive lipids in the jejunum, which are deficient in obese patients, could accelerate the restoration of intestinal functions during weight loss in obese patients.

Apart from oral administration of ELNs, the effect of Shiitake mushroom ELNs intraperitoneally alleviates haemorrhage and cell death in fulminant hepatic failure within 6 hrs administration 56. Pre-treatment with Shiitake ELNs also decreased the serum levels of proinflammatory cytokines like IL-1β, IL-18, and ALT, AST enzyme levels giving promising results for a foot forward to mitigate acute liver injury.

## Role of Edible Exosome-Like Nanoparticles in Gut-Liver Interaction

Various studies suggested that alteration in gut microbiota i.e. gut dysbiosis leads to several diseases. Small intestinal bacterial overgrowth (SIBO) results into production of endotoxins leads to damage intestinal epithelia known as leaky gut and this translocation resulting into hyper inflammation, bacterial peritonitis, hyperdynamic or portal hypertension states. The strategic distal position of liver downstream to gut makes it the most effective organ by the endotoxins or microbiota produced components among other distal organs 57, 58. This could be the main reason that many aetiologies of liver diseases such as liver cirrhosis, NAFLD, alcoholic liver diseases (ALD) including viral hepatitis have different gut microbiota composition 58-60. Even the same has also been seen in autoimmune hepatitis (AIH). Whereas, the numerous metabolites from gut bacteria reaches liver through the portal circulation and takes part in variety of functions 61. Short chain fatty acids (SCFAs) predominantly produced by *Clostridial clusters* IV and XIVa of *Firmicutes* helps in lowering the pH of colon thereby inhibit growth of pathogens 62-65. Butyrate's are also significant source of energy for enterocytes and stimulate mucous production 66. In- vitro it has been shown to decrease the permeability of tight junctions via lipoxygenases hence suggests its probable biological effects on other organs apart from gut 67, 68. Enhancing the production of this metabolite in advanced liver diseases like NAFLD/ NASH using BMEVs could help in reduction of lipid absorption and thereby accumulation in liver to mitigate steatosis 53, 69. *In vivo* studies using OELNs also suggests inhibition in lipid absorption at intestinal epithelia and at liver as well which also makes them perfect candidates to mitigate metabolic syndromes associated to liver diseases 52. Similarly other metabolites that are produced by some other autochtonous bacteria present in gut i.e. *Lactobacillus*, *Bifidobacteria*, *Enterobacter*, *Bacteroides*, *Clostridium* produces bile^70^ or *Faecalibacterium prausnitzii* along with *Bifidobacterium* releases cholines 71 which modulates lipid and glucose homeostasis. As ginger ELNs studies put forward to be successfully shaping the gut microbiota especially the *Lactobacillus* 30 as previously described, it could help in regaining of altered gut which probably help other autochthonous species to colonize in. Lemon ELNs are another probable hope that could be used to get back the gut microbes in a good shape as they acts upon *lactobacillus* and *streptococcus* as well along with push them to produce metabolites which help in inter-bacterial communication in the gut 50. With inhibiting the endotoxins or other components like indole production from *E.coli* they also strengthen the gut immunity and proof their selves as potential therapeutic elements. Hence targeting gut microbiota using ELNs for therapeutic interventions like probiotics could help in developing chronic liver diseases and the metabolic syndromes that are associated to liver. Apart from gut microbes in gut-liver axis, from physiological point of view there exist a strong correlation between High fat Diet and insulin resistance in development of Type 2- Diebetes 72. Cause behind the high consumption of HFD is inactivation of transcription factor (Foxa 2) 73, as activation of the same promotes insulin signalling. Recently in a proof-of-concept study, GELNs showed their potential in prevention of insulin resistance by restoring the expression of Foxa 2 by preventing AKT-1 mediated phosphorylation 74.

## Probiotic and Prebiotic effect during Liver Disease Condition

Interface of gut-liver axis constitute the multilayer defence of physical, humoral and immunological barrier to protect the body from primary exposure of endotoxins and other infectious particles that entered through gut. Liver diseases resulted due to alcoholic and non- alcoholic associated parameters. Alcohol breaches the barrier and this injury compels specific changes in gut microbiota that can enhance alcohol induced liver diseases. Continuous alcohol consumption increases endotoxin producing bacteria and reduction in autochthonous taxa that produces short- chain fatty acids (SCFAs) leads chronic liver disease to cirrhosis and alcoholic hepatitis 75, 76. Besides spectrum of NAFLD ranges from steatosis i.e. deposition of vesicular fat exceeds more than 5% of liver weight and has high prevalence in developed countries like America (21%- 25%), Europe (24%)^77^. Progression of this with hepatocyte ballooning, inflammation, and fibrosis identified as NASH 78, 79. From the extensive studies of gut-liver axis it is undoubtedly proven that healthy gut can leads to a healthy liver. Hence many therapeutic aspects has clinically implemented for making a gut with healthy colonized microbiota in the way to ameliorate progressed liver disease conditions. Living organisms designed for consumption in adequate amounts to get a positive effect on health of host are termed as 'probiotics'. In recent decades some autochthonous microbes like *lactobacillus rhamnassus* GG (LGG) used as probiotics and found to give promising results in recovery of disordered gut hence improving liver disease conditions. Before reaching to their site of action i.e. intestinal region live probiotic bacteria had to tolerate the bactericidal effects of various gastric juices, one of the challenges in applications of probiotics. Their preservation in different climatic zones accordance with transportation also found as obstacles. The gut microbiota varies from one person to another the species of bacteria could be same but the strains varies accordingly. Hence considering probiotics as a common solution cannot be fruitful.

## Modified Edible Exosome-Like Nanoparticles as Nano Drug Delivery System: Future of Treatment

Considering the therapeutic approaches, scientists surmount many challenges mainly safety delivery of therapeutic molecules to reach specific target site, low toxicity and economic production costs. Formation of such cost effective pharmaceutical molecules or nanoparticles with low immunogenicity, cytotoxicity needs complex fabrication processes 80. ELNs are nanosize, innocuous and robust for larger production, represents themselves as a potential candidate to address the aforementioned challenges in translational aspects. Being naturally occurring particles in edible products they have high bioavailability and remain undetected by defence system hence have no immunogenicity. Hence plant derived ELNs have shown their role as nanomedicine and nano-vectors in delivering desired drug molecules and after modification they are termed as nanovesicles.

In development of pristine plant derived nanovesicles (PNVs), the major concern was the preparation of uniform-sized with efficient loading of desired drug molecules to reach specific target site. For which researchers employed the Bligh and Dyer technique based on hydration of a lipid that earlier used for the fabrication of liposomes 10, 81, 82. This liquid-liquid extraction method helps in extraction of membrane lipids and regaining their shape followed by sonication with desirable molecules as drug loading into it. Finally to acquire the uniform- sized vectors with loaded drugs it passes through the liposome extruder with 200 nm polycarbonate membrane 10. At last to check their resemblance into nanosized vectors with spherical shape, their characterization made by using NTA, DLS, TEM along with consideration of their zeta potential.

Aiming to the specificity and biocompatibility with lesser side effects PNVs encapsulated with desired molecules has been implemented against various diseases. It improves the delivery of therapeutic drug molecules that mainly involves hydrophobic drugs, siRNAs, miRNAs or proteins as they easily penetrate into tissues and with an enhanced circulation period. Grape fruit EVs (GfEVs) has been found as efficient carriers for the delivery of exogenously loaded Alexa fluor tagged Bovine Serum Albumin (BSA) and Heat Shock Protein 70 (HSP 70) into Peripheral Blood Mononuclear cells and colon cancer cells. They found as safe, potential and effective carrier for the delivery of exogenous proteins as compared to the proteins without EVs 83.The co-delivery of grape-fruit nanovesicles with folic acid and the chemotherapeutic drug paclitaxel (PTX) showed enhanced specificity and survivability towards colon cancer tumours in xenograft mouse models i.e. CT26 colon cancer and human SW620 colon cancer SCID mouse models by reducing the tumour size 4. These nanovesicles are then attached with receptors, which are inflammatory and leads to activation of leukocytes 10, 84 and 4T1 breast cancer tumours 84 hence extended the survival of tumour-bearing mice. In the same way co- delivery of ginger nanovesicles (GNVs) loaded with curcumin has showed reduction in expression of pro- inflammatory cytokines in DSS induced colitis. Hence they showed the enhanced permeability and retention (EPR) effect of macromolecules and lipids in solid tumours. GNVs along with miR17* in vivo* have seen to be effective against brain GL- 26 brain tumour 35. GNVs incorporated with Incorporating methotrexate (MTX) significantly lowered the DSS- colitis by targeting the intestinal macrophages hence have showed anti- inflammatory responses along with low toxicity as compare to use of MTX alone 85.

## Conclusion

ELNs can be considered as the future of therapeutics specially for the gut and liver diseases. With the advancement of technology, the techniques to isolate, enumerate and characterize makes it much easier to use it as therapy. Not only this, the cargoes information in ELNs, and its association with restoration of gut microbiome homeostasis, hepatoprotective role makes it more potent as treatment option (Fig. 3). With recent trends, the modification of plant derived ELNs into PNVs is safe and easy. But, still many more investigations are required, to take this simple, robust, affordable treatment or drug delivery system to next level for the benefit of patients, suffering with advance gut or liver diseases.

## Figures and Tables

**Table 1:** The edible exosomes, their composition and biological effect

Source of ELNs	Cargos	Effect	References
Ginger	mdo-miR7267-3p	indole-3-acetaldehyde (IAAld)	Teng et al. 2018
	gma-miR396e	Faster growth of LGG	
	ath-miR167a-5p	Prevents LGG Accumulation in Gut Mucosa	
	6-shogaol	Induction of Nrf2, Suppression of TLR4/TRIF	Zhuhang et al. 2015
Garlic	not known	Anti-inflammation, Nontoxicity	Song et al. 2020
Lemon	not known	lactic acid and derivatives	Lei et al. 2020
	not known	Anti-cancerous	Yang et al. 2020
Strawberry	miR166g	Antioxidant Activity	
Rice	MIR168a	Hypercholestromia	Zhang et al. 2012
Broccoli	Sulforaphne	Anti-inflamatory, activates AMPK patway	Deng et al., 2017
Grape fruit	not known	Anti-inflammatory	Teng et al. 2018
Arabidopsis thaliana	ath-miR159a	Anti- tumor effect in breast cancer	Wu et al. 2016
Soybean (Glycine max)	gma-miR159a-3p, gma-miR159e-3p	Anti- tumor effect in breast cancer	Wu et al. 2016
Dendropanax morbifera Leaf derived Evs (LEVs)	not known	Anti-melanin	Lee et al. 2019

**Table 2:** The clinical trials undergoing presently for liver diseases

Edible exosomes	Associated with therapy	ClinicalTrials.govt
Grape-derived	Oral mucositis (associated with chemoradiation therapy)	NCT01668849
Aloe- and ginger-derived	Treatment and migration of symptoms (e.g., insulin resistance and chronic inflammation) associated with polycystic ovary syndrome (PCOS)	NCT03493984
Curcumin for delivery	Colon tumor	NCT01294072

## Abbreviations

ELNs: Edible Exosome-Like Nanoparticles
ALD: Alcoholic liver disease
NAFLD: Non-Alcoholic liver disease
PDEVs: Plant Derived Extracellular Vesicle
Evs: Extracellular Vesicle
PELNs: Plant derived exosome-like nanoparticles
UC: Ultracentrifugation
PEG: Polyethene glycol
DEX: Dextran
ATPS: Aqueous two-phase system
TEM: Transmission Electron Microscopy
SEM: Scanning Electron Microscopy
AFM: Atomic force Microscopy
DLS: Dynamic Light Scattering
NTA: Nanoparticle Tracking Assay
PA: phosphatidic acid
PC: phosphatidylcholine
PE: phosphatidylethanolamine
AELNs: Acerola Exosome-Like Nanoparticles
GELNs: Ginger Exosome-Like Nanoparticles
GrELNs: Garlic Exosome-Like Nanoparticles
GfELNs: Grapefruit Exosome-Like Nanoparticles
OELNs: Orange Exosome-Like Nanoparticles
PNVs: Plant derived Nanovesicles
GfEVs: Grape fruit derived Extracellular Vesicles
BSA: Bovine Serum Albumin
HSP 70: Heat Shock Protein 70
GfNVs: Grapefruit Nanovesicles
GNVs: Ginger Nanovesicles
SCFAs: short chain fatty acids
